# 

**Published:** 1896-10

**Authors:** 


THE British Medical Association will meet in Montreal, Canada, the last week in August, 1897. This will be a memorable occasion as being the first time in the history of the Association for the meeting to be held elsewhere than in one of the cities of the British Isles. No doubt hundreds of physicians from the United States will attend the meeting, while other hundreds from Britain will be in attendance at the meeting of the American Medical Association in Philadelphia, in June, 1897. It is a pleasure to announce that the proceedings of both of these great Associations are conducted in the English language.

### CONTENTS.

THE DENTAL REGISTER,

A MONTHLY MAGAZINE DEVOTED TO THE INTERESTS OF THE

**DENTAL PROFESSION.**

*Terms Reduced to $2.00 Per Year. Single Copies, 25 Cents.*

**EDITED BY PROF. J. TAFT, D.D.S.**

AND

**Published by SAM'L A. CROCKER & CO., Ohio Dental and Surgical Depot.**

The volume commences in January and closes in December.

The REGISTER being issued monthly is always fresh, therefore Indispensable to the practitioner who desires to keep posted up with the new inventions and improvements which are daily developed. Neither expense nor effort will be spared to give value and variety to its contents. Articles will be illustrated with well executed engravings whenever they will add to the interest or assist to explanation.

Its extensive circulation and frequency of issue make it one of the BEST DENTAL ADVERTISING MEDIUMS in the United States.

All subscriptions must begin with January or July.

Local or special notices, five lines or less, will be inserted for $3 each insertion; over five lines, 50 cts. per line.

All advertising bills payable in advance, or at furthest, after the issue of the first number containing the advertisement. All transient advertisements to be paid for in advance.

☞CONTRIBUTIONS SOLICITED.

Exclusively Editorial Communications should be addressed to the Editor.

The latest number of the REGISTER may be ordered with or without other good [illegible] any time, and all letters should be addre[illegible]

